# Empowering Energy Justice

**DOI:** 10.3390/ijerph13090926

**Published:** 2016-09-21

**Authors:** Mary Finley-Brook, Erica L. Holloman

**Affiliations:** 1 Department of Geography and the Environment, University of Richmond, Richmond, VA 23173, USA; 2 Southeast CARE Coalition, Newport News, VA 23607, USA; holloman.e.l@gmail.com

**Keywords:** energy justice, energy transitions, climate justice, participation

## Abstract

The U.S. is experiencing unprecedented movement away from coal and, to a lesser degree, oil. Burdened low-income communities and people of color could experience health benefits from reductions in air and water pollution, yet these same groups could suffer harm if transitions lack broad public input or if policies prioritize elite or corporate interests. This paper highlights how U.S. energy transitions build from, and contribute to, environmental injustices. Energy justice requires not only ending disproportionate harm, it also entails involvement in the design of solutions and fair distribution of benefits, such as green jobs and clean air. To what extent does the confluence of state, civic, and market processes assure “just” transitions to clean, low-carbon energy production involving equitable distribution of costs, benefits, and decision-making power? To explore this question we assess trends with (1) fossil fuel divestment; (2) carbon taxes and social cost of carbon measurements; (3) cap-and-trade; (4) renewable energy; and (5) energy efficiency. Current research demonstrates opportunities and pitfalls in each area with mixed or partial energy justice consequences, leading to our call for greater attention to the specifics of *distributive justice*, *procedural justice*, and *recognition justice* in research, policy, and action. Illustrative energy transition case studies suggest the feasibility and benefit of empowering approaches, but also indicate there can be conflict between “green” and “just”, as evident though stark inequities in clean energy initiatives. To identify positive pathways forward, we compile priorities for an energy justice research agenda based on interactive and participatory practices aligning advocacy, activism, and academics.

## 1. “Just” Energy Transitions

Movement away from high carbon energy sources like coal and oil is essential to contain climate disruption, reduce pollution, and create important health improvements [1,2,3]. As the U.S. adopts lower carbon options, widespread race and class inequalities persist. Without attention to power and resource inequalities, tensions between “green” and “just” will continue [2,4,5]. “Just” environmental governance is inclusive, recognizes low-income populations and people of color are important sources of knowledge and solutions, and involves impacted communities in the planning and redevelopment of healthy spaces [6].

**Figure 1:**
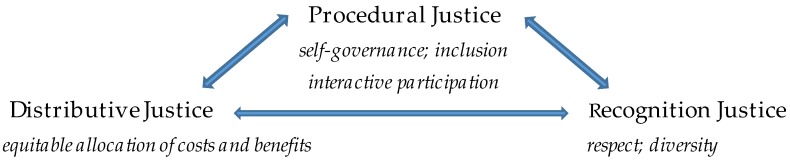
Energy justice.

Energy justice (Figure 1) requires: *Distributive justice* with equitable allocation of risks and opportunities; *procedural justice* with access to decision-making power; and *recognition justice* involving respect for all peoples and worldviews [7]. With significant energy transitions underway, and yet disappointing justice implications thus far, this paper calls for empirical and participatory research with attention to all three tenets of energy justice (procedural, distributive, recognition) as a means to inform policy and action. We emphasize the importance of procedural decision-making power to facilitate gains in the other two areas. Effective participation of low-income communities and people of color in energy decision-making can be a catalyst for broader change. Unfortunately, civic involvement today occurs on a broad spectrum and there are more examples that would be categorized as ineffective (i.e., passive or manipulated) than effective (i.e., involving interaction and empowerment) [8].

Energy justice (Figure 1) is an instrumental frame for research and activism alike. Using interdisciplinary methods, energy justice researchers seek to identify where injustices are, who is impacted and how, and what remedies exist [9,10]. Filtering energy issues from broader climate justice (CJ) and environmental justice (EJ) contexts can aid in the formulation of appropriate energy solutions and may help identify and build social mobilization for energy transitions. While energy justice is not widely used as an activist frame, and does not have activist origins like EJ, the concept has the potential to frame and bolster activism and advocacy [9,10,11].

Combining constructs of *energy justice* with *systems thinking* allows identification of linkages among all energy system components, connections, and processes from start to finish, essentially a life cycle analysis. This framework allows for attention to (1) the triumvirate of justice concerns (distributive, procedural, recognition) [12]; (2) linkages between sites of production and sites of consumption clarified by analyzing processes from extraction through processing, transportation, and consumption to waste disposal; and (3) energy as a socio-technological assemblage of human and non-human agents, places, and processes. It is important to acknowledge the role of non-human elements, such as roads, pipelines, generating plants, and power lines, because of the enormous psychological, physical, and financial influence that landscapes and carbonscapes have on future energy expectations and decisions [13].

Section 1 contextualizes climate and energy justice as EJ issues. Section 2 highlights how the inadequacy of federal policies and programs spurs pressure for change. Section 3 assesses the equity and justice potential of greenhouse gas (GHG) reduction (decarbonization) strategies. Section 4 provides examples of transformative community initiatives, while also pointing out on-going tensions and paradigmatic mismatches in environmental networks. Section 5 recommends priorities for an energy justice research agenda. Our focus is limited to the U.S., but our findings may have implications further afield.

### 1.1. Climate Justice

Significant action to curb GHG emissions is necessary to avoid an estimated trillion dollars in property and ecosystem damage as well as sharp increases in hunger, migration, and conflict [14]. Due to economic and political marginalization, people of color could be less able to respond to extreme weather, changes to water availability, or food insecurity [14,15,16]. Native Americans in Louisiana and Alaska are the first U.S. climate refugees, while coastal areas experiencing sea-level rise, including locations surrounding Miami, New Orleans, New York City, and Hampton Roads, host concentrations of African Americans and Latinos.

The 2015 United Nations (UN) climate negotiations led to a Paris Agreement signed by 174 parties; however, implementation is voluntary and options for GHG reduction are flexible. Past experience with UN climate change mitigation suggests that instead of targeting deep or systemic transformation in domestic sectors wealthy countries often purchase low-cost transnational offsets [17,18,19]. While human rights are mentioned briefly in the Paris Agreement’s preamble, there are no specific norms assuring the protection of human rights, which is worrisome because some past carbon offset projects have spurred negative social outcomes and violent repression [16,17,18,19]. Climate change mitigation and adaptation projects and policies must respect the rights of local populations, including water, resource, worker, and cultural rights. Focusing on reduction of GHG emissions in isolation from CJ frameworks is likely to worsen inequality and could harm marginalized and vulnerable populations, including women, children, elderly, disabled, people of color, and low-income groups [2,4,18].

Redistributive outcomes and participatory decision-making in climate policy are challenging given historical injustices and differences in goals, priorities, and perspectives among stakeholders. Even when attention to equity and justice occurs, there can still be a chasm between stated intentions and implementation realities due to entrenched departures in values, methods, and objectives [20]. As a case in point, market-based “green” initiatives are frequently criticized in EJ and CJ circles because they limit the power and agency of low-income populations, such as when cap-and-trade programs allow for carbon offsets in distant locations in exchange for the right to pollute in burdened EJ communities [18,20,21].

### 1.2. Energy Justice

Benefits from centralized energy systems are usually poorly distributed, meaning that change threatens interest groups gaining from the status quo [22]. Fossil fuel corporations help shape U.S. energy policies [23], in part because these heavily capitalized firms employ powerful lobbyists, thus influencing the energy transition options policy-makers consider [24]. As an example, coal and oil companies have spent millions of dollars lobbying against distributed renewable energy [23].

Geographic and spatial factors influence energy justice because concerns are frequently tied to detriment linked to specific sites of extraction, refining, transportation, storage, combustion, consumption, or waste disposal. Negative impacts from the energy sector, such as air pollution, are often unevenly distributed. Young children and unborn fetuses are particularly prone to harm from pollution from fossil fuels [25], which includes particular matter, polycyclic aromatic hydrocarbons, nitrogen oxides, sulfur dioxide, and volatile organic compounds [21,22,25]. When Perera [25] calls for a child-centered revamping of U.S. energy policy, she recognizes children of color and those living in low-income communities are disproportionately impaired by contamination. Negative health impacts can intensify without insurance or affordable medical care [26]. EJ communities are more likely to experience premature death and debilitating cardiovascular and respiratory disease due to workplace and residential exposures [1,24]. Disadvantaged neighborhoods tend to host refineries, power plants, transportation corridors, ports, and other industrial land uses producing high emissions [27]. Over 70% of African Americans live in counties that violate federal air pollution standards [28]; moreover, air monitoring systems may not capture hotspots and thus misrepresent pollutant levels [21,29].

U.S. coal production is at the lowest level in three decades. While resulting public health gains could be high, economically and politically marginalized communities could also experience negative impacts, such as rate hikes or loss of employment, when their needs and rights are not taken into consideration during energy transitions [2,4]. Mine, refinery, and plant closures can harm households dependent on fossil fuel jobs [4,22]. In 2013 the Navajo Nation bought a coal mine with the objective to protect the local economy and tribal sovereignty [30]. Today, demonstrating a significant shift, the Navajo-run Black Mesa Water Coalition is turning a former mine site into a community-run solar farm.

Global wind and solar installations grew by 30% in 2015. The U.S. added 7 gigawatts of solar, but benefits from this boom were not distributed equitably. African Americans hold fewer solar jobs than other racial and ethnic groups [31]. Rooftop solar panels and energy efficiency upgrades leading to savings on utility costs are more likely to benefit wealthy homeowners than low-income renters [4,32]. Energy transitions highlighting efficiency and cost-effectiveness can reinforce existing social imbalances: In the green energy sector, wealthier populations are more likely to gain, sometimes at the expense of the poor [32].

## 2. State and Civic Actions towards Energy Transitions

Environmental justice initiatives received more attention and resources under President Barack Obama and yet subsequent changes to the energy sector have been slow. Dissatisfaction with fossil fuel pollution encourages public pressure on federal agencies to accelerate energy transitions [33,34,35].

### 2.1. Federal Policies and Programs

Coal, oil, and gas firms receive special treatment under federal laws, including exclusions, exemptions, and grandfather clauses, meaning safe air and water pollution limits have regularly been surpassed [22]. As the Environmental Protection Agency (EPA) increases efforts to reduce energy sector pollution, it has received backlash from various members of the U.S. Congress [21,22]. While improving monitoring, the EPA remains unable to enforce compliance at every power plant, in part due to budgetary constraints. Nevertheless, the Toxic Release Inventory website and other EPA databases increase transparency. The Emissions and Generation Resource Integrated Database shares environmental characteristics of different types of power generation, while the Power Profiler provides data on fuel types and emissions by zip code and as compared to national averages.

Although there has been creation of new federal programs to support energy transitions, concerns exist about access for those most in need as well as criticisms of limitations to participation in planning and decisions. The EPA’s Clean Energy Incentive Program (CEIP) provides grants for solar, wind, geothermal, hydropower, and energy efficiency projects in low-income communities, but states must commit matching funds, and some have not taken advantage of this program. EJ concerns regarding the CEIP are that (1) the main criteria for defining eligibility is income and the issue of race has been excluded; (2) state agencies are granted flexibility to define “low-income” populations and to determine how they participate in decision-making, which in the past has not assured meaningful involvement of marginalized or vulnerable groups; and (3) the CEIP incentivizes energy efficiency programs through Renewable Energy Credits and Allowances granted to state agencies, rather than direct transfers to low-income communities [36].

Other examples of support for energy transitions include a federal Economic Development Agency program for coal workers to receive training for new jobs. The Department of Energy’s community solar program seeks to expand access for occupants of multi-unit housing or high rises, while the Weatherization Assistance Program assists low-income households with efficiency upgrades. Although these initiatives are important, current levels of support do not adequately address energy insecurity among the poor. Fatalities during extreme cold or heat waves result from vulnerable populations not being able to afford electricity [15]. Low-income families sometimes have to choose between buying food and paying for utilities.

In 2004 President Bill Clinton’s Executive Order (EO) 12898 brought to the surface the depth of environmental injustices in the U.S., but progress from EO 12898 was limited due to the lack of enforceable statutes [5]. Two decades later the EPA’s EJ Plan 2014 created a roadmap to integrate justice broadly into EPA work, to empower communities to become engaged in decision-making, and to improve scientific tools and informational resources to support EJ outcomes [5,20]. Nonetheless, the National Environmental Justice Advisory Council expressed concerns about the plan’s lack of clear goals or specific timeframes and highlighted the need to codify norms, so that EJ objectives are understood as obligations and responsibilities [37].

While EJ Plan 2014 has not generated enforceable rules, new tools and programs like EJSCREEN, a geospatial analytical tool that cross-references demographic and environmental data, help identify EJ locations that merit additional attention [5]. Under the “Making a Visible Difference” program, the EPA works with “environmentally overburdened, underserved, and economically distressed areas” committing technical and financial resources in all 50 states [38]. However, there are still many communities experiencing high-level environmental injustice not covered in this pilot. Furthermore, while seeking to assure community members are consulted, the agency continues to works more closely with state and local governments than with grassroots organizations and to operationalize a relatively top-down model for change.

While the EPA faces impediments to address the extent of environmental injustice in the energy sector, the addition of a Senior Advisor on Environmental Justice for the EPA Administrator suggests continued attention to EJ concerns in spite of financial and political limitations placed on the agency. Nevertheless, analysis of federal energy governance demonstrates progress toward procedural justice remains slow. A series of specific EPA programs targeting areas with high toxic burdens do exist, but participatory decision-making continues to be challenging and communities located throughout the country suggest energy injustices they experience have not been recognized or remediated [39,40,41].

### 2.2. Activism and Increasing Pressure for Energy Transitions

With the federal government unable or unwilling to limit fossil fuel pollution, public pressure for change is intensifying [15,33,39,40,41]. Community organizing often starts as a response to maldistribution of risks and harm. Each local concern usually relates to a different part of an energy system (e.g., extraction, refining, transportation, storage, waste disposal, etc.). The localized nature of EJ concerns and movements is a motivational and organizational strength, yet can potentially be a weakness as well if there is fragmentation between locations and groups. As an illustrative example, African American residents of Uniontown, Alabama are working with lawyers to demand remediation following extensive coal ash disposal in a landfill in their neighborhood. Understandably, Uniontown residents are working to lower health risks and reduce environmental damage in their own community, rather than trying to impede coal combustion and pollution altogether. However, feelings of solidarity among impacted communities are common. Identifying connections between sites of impact can fortify cooperation and strengthen networks for deeper economic and environmental transformations. Peer-to-peer exchanges and training programs build dialogue and avoid splintering in EJ advocacy.

**Figure 2:**
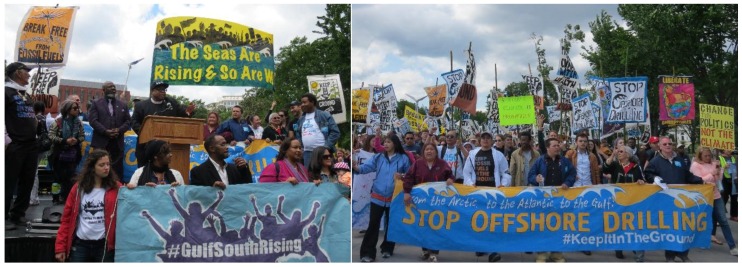
“Break Free” Protests in Washington, DC (Photo credits: Mary Finley-Brook).

Anti-fossil fuel protests are occurring throughout the world with increasing frequency. National and international networks organizing and participating in these rallies generally include a relatively limited number of EJ and CJ groups amidst many non-governmental organizations (NGOs), faith-based groups, and concerned citizens motivated to take a proactive role in determining the speed and direction of energy transitions. “Break Free” actions in May of 2016 involved protests on six continents, including blockades at several coal and gas sites leading to arrests. In Washington, DC, frontline communities from the Arctic, Atlantic, and Gulf (Figure 2) led a rally and protest march to denounce offshore oil drilling and sea-level rise, stating “the seas are rising and so are we”. This phrase expresses momentum toward and desire for procedural justice.

According to survey data, EJ organizations increasingly recognize they have not focused on climate action sufficiently but are intensifying their efforts moving forward [42]. EJ networks and groups leading climate-oriented work include the Environmental Justice Leadership Forum on Climate Change, WE ACT, the Environmental Justice and Climate Change Initiative, the Just Transition Alliance, the Indigenous Environmental Network, and Texas Environmental Justice Advocacy Services, among others. The 2016 Public Policy Agenda from a network of national Hispanic organizations includes climate justice for the first time. A growing number of alliances are emerging between EJ, CJ, racial justice, labor, and immigrant rights movements. However, broad alliances can translate into multi-faceted demands with climate and energy injustices making up only a small segment of interrelated concerns.

As covered in Section 3, civic engagement has become part of decarbonization strategies, such as with divestment campaigns or community solar. Alternatively, public engagement can also occur as an oppositional response to a decarbonization strategy, such as with cap-and-trade. Energy transitions are not merely about using different types of fuel, but also require complex, interrelated changes in the social, economic, and political arrangements established in conjunction with energy technologies [24]. Disagreement over the scope of changes perceived as necessary can create a paradigmatic rift, as addressed in Section 4. Some environmental groups suggest that the economy is generally working fine and the specific problem that needs to be fixed is the release of excessive GHGs [18]. In contrast, EJ and CJ movements aim for fundamental restructuring of the economy to increase economic and social equity and promote community empowerment, health, and resiliency [16,18,33]. To do this, they seek to integrate distributive, procedural, and recognition justice.

## 3. Equity and Justice in Decarbonization Strategies

In subsequent sections, we assess five strategies for decarbonization: Divestment, carbon tax/social cost of carbon measurements, cap-and-trade, renewable energy, and energy efficiency. We identify areas of energy justice (distributive, procedural, recognition) relevant to each strategy along with risks and opportunities for implementation.

### 3.1. Fossil Fuel Divestment

Although fossil fuel divestment is relatively new, it has already been identified as an “important catalyst” of an “energy revolution” [34] (p. 2). Since the initiation of fossil fuel divestment movements in 2011, major financial and political leaders have made public statements in support of leaving remaining hydrocarbon resources in the ground [43]. Over 530 institutions, approximately half of these located in the U.S., have approved divesture of $3.4 trillion [44]. Institutions divesting include faith-based groups (27%), foundations (24%), governmental organizations (14%), educational institutions (13%), pension funds (13%), NGOs (6%), for-profit corporations (3%), and health organizations (1%). In addition, approximately 50,000 individual investors have divested $5.2 billion.

Divestment occurs by selling assets linked to specific firms or sectors. As divestment gains momentum, perceptions of risk from carbon exposure and stranded assets reinforces preference for lower carbon energy investments [34,45]. Divestment Strategies 1–6 in Table 1 are relatively common while Strategies 7 and 8 are not currently utilized; nevertheless, we suggest these provide opportunities to improve social justice impacts. Strategy 7 would alienate firms identified to have significant and disproportionate impact on low-income households or people of color, as determined through demographic analysis near high-polluting energy facilities. Strategy 8 would target firms with recurring violations of worker rights or environmental laws. Notably, there is often overlap among offenders in Strategies 5–8. Another means to improve social justice from divestment is to improve reinvestment to replace lost jobs (Strategy 9). Few campaigns target divested funds directly at green sector employment, although options exist with divest-invest philanthropy [46]. The Movement for Black Lives (made up of 50 organizations, including Black Lives Matter) takes a broader approach to divest-invest, seeking fossil fuel divestment along with decriminalization through reduction of funds for incarceration, prison systems, and surveillance [47]. They argue funds should be re-invested in health, education, job training, and public safety.

It is too early to document fossil fuel divestment movement impacts, although Table 2 lists potential direct and indirect consequences. Experts suggest that divestment campaigns are more effective at communicating a symbolic message to an economic sector than damaging specific firms. We recommend including justice as a core component of divestment strategies, although each investor would be responsible for defining what qualifies. While claims to morality often motivate divestment advocates [34,45], EJ concerns have seldom been forefront. The majority of campaigners are white, although the Divestment Student Network’s People of Color Caucus [48] supports members of color and promotes diverse participation. As shown in Table 2 divestment has the potential to support distributive, procedural, and recognition justice, but to avoid risks we recommend additional focus on economic justice, decision-making power, rights, and diversity.

### 3.2. Carbon Pricing: Taxes and Social Cost of Carbon

By 2015, 14 countries had passed carbon tax legislation and 18 were taking steps toward carbon pricing readiness [49]. Emissions reductions occur because taxes create price increases for high-carbon industries and activities, thus lowering demand and encouraging fuel-switching to less carbon-intensive options [50]. There is growing attention to carbon taxes among environmentally and economically-focused NGOs as a response to the fact that current state carbon policies are inadequate.

Carbon pricing has positive and negative equity impacts (Table 3). Low-income groups are likely to be harmed by price increases and so redistributive procedures are necessary to minimize negative impacts [51]. There are several options for equity weighting, such as direct payments, tax rebates, or state assistance programs to help pay fuel bills or weatherization costs. However, the concept of fairness could become thorny; for example, an argument could be made that people living in rural areas, or those with long commutes to and from work, or without access to public transport, or living in particularly hot or cold climates should be compensated for higher fuel expenses.

To improve EJ implications from this strategy, we suggest additional attention to potential risks from carbon pricing along with efforts to broaden decision-making. More accurate market signals may spur energy transitions, but some price uncertainty continues since it can be difficult to pinpoint discount rates or the costs of impacts such as social unrest [49,52]. Nonetheless, intergenerational equity is likely to improve following a carbon tax, since emission reductions today would reduce future harm [50]. Yet, since GHG emissions linger in the atmosphere, an underlying assumption is that there will be harm now and into the future from past releases, but no specific emitter is held financially accountable.

The social cost of carbon is a comprehensive estimate of complex repercussions from climate change such as disruptions in food production and human health impacts [52,54]. These costs are currently taken into account in cost-benefit analysis of some federal regulations; for example, agencies like the EPA measure the benefits of regulations that reduce emissions and weigh them against the costs of the regulation. Given our growing understanding of these social costs, the fact that they are not more routinely calculated or more broadly applied suggests undervaluation of social harm that is likely to be felt inequitably by vulnerable groups.

### 3.3. Cap-and-Trade

Prominent cases of cap-and-trade include the European Union Emissions Trading System, the Western Climate Initiative, and the Regional Greenhouse Gas Initiative. Cap-and-trade is popular with some policy-makers because it is perceived as cost-effective and flexible, particularly when transboundary trade allows emitters to chase lowest cost offsets in other locations rather than making more expensive reductions within the same geographic region. Cap-and-trade is a distribution-oriented policy with limited opportunities for procedural or recognition justice; nonetheless, EJ organizations have worked hard to promote positive outcomes and avoid risks and burdens in places like California experimenting with emissions trade. Although trading systems provide an overall cap, they have been criticized for not reducing emissions aggressively enough and for being unfair because they allow those with resources to pay to pollute, while potentially expanding toxic burdens for disadvantaged groups (Table 4) [18,21,55].

The California Global Warming Solutions Act of 2006 (AB 32) combines a renewable portfolio standard for electricity, a low-carbon fuel standard for transportation, and GHG cap-and-trade [53,56]. The cap-and-trade portion of AB 32 became a lightning rod for controversy, in part due to differing valuation of benefits and risks: Some focus on the potential to increase the toxic loads in EJ communities, while others applaud the chance for broad benefits from the overall reduction of carbon and other co-pollutants, such as sulfur dioxide and nitrogen oxides [18,56]. Even after EJ organizations participated in the drafting of AB 32, conflict during implementation lead to a 2009 lawsuit pitting EJ advocates known as the Association of Irritated Residents against California’s Air Resources Board [59]. The lawsuit was unsuccessful, although many EJ activists remain critical of the state agency’s lack of procedural transparency and what some considered to be rushed assessments of implementation alternatives [19,54,57,58,59,60,61].

In 2012, Senate Bill 535 followed AB 32 to mandate that 25% of cap-and-trade funds benefit “disadvantaged communities” and that at least 10% of these funds should be invested directly in such areas [57]. Nevertheless, a persistent critique is that the existence of these funds serves as a distraction from the fact that EJ communities did not support the market-based mechanism in the first place [61]. Furthermore, many EJ advocates remain disappointed with divergence from initial AB 32 promises [20].

The California Environmental Protection Agency identifies “disadvantaged communities” to received AB 32 benefits using the California Communities Environmental Health Screening Tool and the Environmental Justice Screening Method. Both incorporate multiple data sources to calculate an overall cumulative impact score and both have been developed in collaboration between scientists and EJ activists, who continue to engage in the AB 32 process to try to influence positive EJ outcomes [58,61]. Computer screening tools are cost-effective and rapid in determining multiple risks and exposures, but it is important to make sure training is available so technology does not become a barrier. In California, community members have participated in hands-on data collection to validate secondary databases [62], encouraging transparency and the co-production of knowledge.

Californian cap-and-trade receives significant attention, but the process is relatively new and it is too soon to record results. The state provides a unique test case due to the presence of exceptionally strong EJ organizations when compared to other states. Distributive, procedural, and recognition justice in cap-and-trade programs in other parts of the U.S. and Canada remain relatively poorly understood, although international research on emissions trade is extensive and suggests mixed results involving successes and failures. Researchers frequently document examples of exclusion, displacement, and inequality [16,18,33]. Akin to international offsets, Californian processes suggest the need to follow changes across time and space. Trade takes years to implement and additional time is necessary to record measurable impacts, which are likely to play out differently in specific locations.

### 3.4. Renewable Energy

Renewable portfolio standards (RPS) with requirements for renewable sources exist in more than half of U.S. states; however, some states use broad definitions of “renewable”, accepting “clean” coal, nuclear, and waste incineration, among other technologies [63]. States consider a broad range of factors when determining fuel types and other RPS targets, but do not broadly consider social impacts when advocating for or against different options.

The costs of renewables have declined in recent years, creating economic opportunities in urban and rural areas alike (Table 5). Although commercial-scale solar installations replace fossil fuel plants most rapidly and efficiently, some large installations utilize vast land areas and compete with other conservation or production uses. Community-based models might be considered less cost-effective based on conventional economic calculations, but they provide additional benefits, such as social transformation, empowerment, and small-scale or collective entrepreneurship [64].

After several years of rapid growth, the U.S. solar industry already employed 77% more workers than the coal industry in 2015 [31]. African American workers are largely being left out of the U.S. solar boom, holding only 5.2% of positions in 2015 even though they make up 11.7% of the U.S. workforce. Latinos are also underrepresented with 11.3% of solar jobs while Asians and Pacific Islanders are overrepresented at 8.7%.

In California, where solar development has outpaced the rest of the country, commercial solar installation jobs often involve a five-year apprentice training programs to produce skilled professionals earning competitive salaries and benefits. In contrast, a dual labor market seems to be emerging with rooftop installations whereby relatively untrained short-term laborers are recruited from temporary work agencies or by contractors to earn low hourly wages without benefits [65]. While there are numerous stories of non-profit solar training programs positively changing the lives of marginalized workers, including unemployed youth and those formerly incarcerated, a portion of businesses in California’s giant unregulated solar market appear to profit from the exploitation of unskilled laborers. This pattern requires additional research and policy attention as rooftop programs expand throughout the U.S.

To assure access to skilled positions in the renewable energy sector will require improved science, technology, engineering, and mathematics education for underserved populations. Similarly, in terms of purchasing solar, higher household incomes and higher education levels lead to increased use, while lower household incomes and higher housing density reduce adoption rates [66]. Homeowners have the advantage of using collateral to obtain home equity or consumer loans, meaning rooftop solar is often perceived as more accessible to the middle and upper classes. With net metering, unused solar energy provides credit towards energy bills, meaning savings can be considerable. Virtual net metering for community-owned projects has the potential to bring solar benefits to low-income populations who do not own their homes. Community projects can also provide shade for parking lots, be placed on transportation infrastructure, or serve as solar “gardens” in public spaces [35].

Solar microgrids are increasingly popular as a method to increase urban resiliency. Yet, instead of establishing year-round systems, local governments often invest millions of dollars into short-term emergency back-up systems for use following natural disasters, while keeping daily generation tied to fossil fuels. Two important constraints to microgrid innovation appear to be (1) long-term contracts with large utility companies binding state agencies and other institutions to traditional systems, and (2) narrow cost-benefit calculations citing price per kilowatt hour while ignoring social gains, including opportunities for community engagement and health benefits from pollution abatement.

### 3.5. Energy Efficiency

Energy efficiency is the most cost-effective method to reduce GHG emissions and has significant positive repercussions for energy justice (Table 6) [67]. Given historical patterns of inequality in siting energy plants, such that they are disproportionately located in low-income communities of color, there are potential EJ implications if gains in efficiency can reduce the need for construction of new energy facilities. Since electricity costs make up a higher percentage of low-income household budgets, efficiency programs provide greater relative benefits to poor households [15,21]. In addition, energy efficiency programs frequently form part of multi-faceted community re-development efforts to increase education and build empowerment while improving health and safety.

Energy efficiency improvements can remain out of reach to those unable to pay upfront costs or who do not own their residences [68,70]. Landlords are less likely to invest in weatherization than homeowners, and low-income housing units, particularly those held by absentee landlords, are some of the most in need of efficiency upgrades. An under-utilized solution for renters is on-bill financing allowing payment for upgrades in monthly installments made more affordable by reductions in energy bills as a result of efficiency improvements [35]. Another option to reach the neediest sectors would be for Section 8, the state voucher program to assist low-income families, the elderly, and the disabled obtain affordable housing, to require basic weatherization and energy efficiency standards, with technical and financial assistance where necessary.

### 3.6. Comparison of Decarbonization and Justice Types

Although there have been rapid energy shifts in a few select areas and sectors, such as with the growth of solar energy in California, energy transitions generally occur slowly. Our research on five decarbonization strategies, depicted in Table 2, Table 3, Table 4, Table 5 and Table 6, suggests that risks are widespread while opportunities are uncertain for marginalized populations. All decarbonization strategies appear prone to manipulation through political interference or industry influence. Within the strategies we assessed, most attention seems to have been granted to distributive concerns, while recognition justice appears to receive least attention.

**Figure 3:**
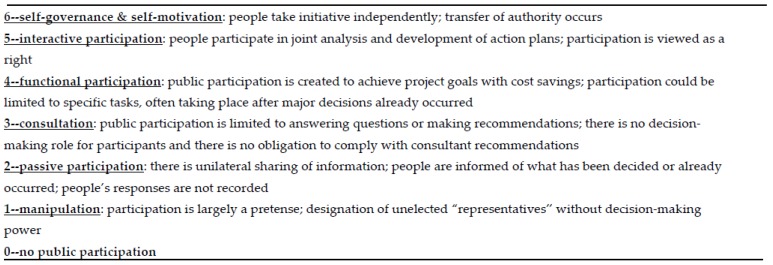
Typology of participation [8]. Modified with permission from J. Pretty, *World Development*; published by Elsevier, 1995.

Our research findings suggest restrictions to authentic participation impede the actualization of energy justice. Figure 3 shows a typology of participation and points out tendencies in the bottom levels aimed at limiting decision-making power while creating the pretense of participation. To achieve authentic participation, anything less than level five in the typology is inadequate. In the literature we reviewed it was rare to find examples where interactive, empowering, and diverse participation occurred if state institutions or organizations located outside of a local community facilitated energy sector interactions.

## 4. Successes, Tensions, and Next Research Steps

Without waiting for state or market shifts, grassroots organizations create positive change. With more than 30 years of experience, and after establishment of regional offices in different parts of California, the EJ NGO Communities for a Better Environment (CBE) connects racial injustice at one energy production stage or site, such as the location of oil refining, with racial injustice at another, such as the movement of oil trains (colloquially known as oil “bombs” due to the risk of explosion) through densely inhabited communities of color. With a proven track record of influencing state policy, CBE combines public education and participatory action research with protest and litigation. CBE is not alone in its success. Far from exhaustive, the following list includes noteworthy efforts in locations throughout the U.S. (Table 7). Positive cases like these seldom receive the research attention they deserve, as there is much to be learned from transformative community-run successes.

While Table 7 illustrates important progress, areas of entrenched injustice remain where energy transitions have not occurred. Neighborhoods in California, Illinois, Michigan, and Texas, among other states, have fought for years, and sometimes decades, to close or clean up coal plants and petroleum refineries. Many times plants were out-of-compliance or used outdated pollution control equipment.

Transition away from dirty energy is slow and communities living nearby facilities often express feelings of exclusion from decisions about site remediation. Little Village Environmental Justice Organization (LVEJO) worked for more than a decade to close two coal plants in their Chicago neighborhood. Following victory in one stage with plant closure, LVEJO seeks involvement in the next stage with an alternative energy project to transform the site contaminated with mercury, arsenic, and other toxic substances [71]. LVEJO’s long-term commitment to this neighborhood allows members to proactively seek solutions, thus moving beyond criticizing harm, which was the necessary focus of the movement at the beginning. While LVEJO networks with national groups to collaborate on broader campaigns, the focus of staff and volunteers remains in Little Village, where long-term neighborhood transformation inspires grassroots struggles for change in other locations.

To learn from energy justice governance in the cases in Table 7 requires additional attention to specific procedures. Another stage of analysis could assess if and how these types of initiatives might apply to new locations while respecting local differences. The success of these local-level efforts is encouraging, but it is readily apparent that there is disjuncture between the scale and scope of these solutions and the impacts of large energy firms. Fossil fuel use remains entrenched and dominant. Although coal use is diminishing, oil and gas infrastructure is still expanding, as seen through dozens of multi-million dollar pipelines in planning and construction stages. These megaprojects are frequently contested, such as with the proposed construction of the Dakota Access Pipeline: The Standing Rock Sioux organized the largest Native American protest in recent history to shed light on the destruction of burial grounds and spiritual sites as well as the need to protect tribal land and water resources from potential spills during crude oil transportation.

Researchers must continue to document and analyze new and emerging risks. What role does the development of hydraulic fracturing, or fracking, play? How does decline or expansion of extraction in one area or energy type influence broader processes? Do historical patterns from oil and coal industries continue with natural gas? All three fuel industries harm the health of Native American populations in Oklahoma [72]. Research in south Texas suggests people of color, particularly Latinos, disproportionately live near injection wells for fracking waste [73]. EJ research linked to natural gas production and transport is likely to increase, as coverage to date has been relatively limited when compared to other fossil fuels.

Energy injustice associated with the transportation sector and mobile energy sources requires additional attention. Cumulative impacts of pollutants from multiple sources are increasingly recognized and measured. In southeast Newport News, Virginia, an area receiving EPA’s “Making a Visible Difference” assistance, a predominately African American neighborhood lives with cumulative exposures from coal export terminals, highways, a naval shipyard, and industrial corridors. Documenting how fossil fuels are key to each of these facilities makes it clear that energy transitions remain scarce in this community.

### Existing Tensions and Potential Conflicts

It is important to acknowledge that there continue to be tensions that threaten positive collaboration in energy transition networks. Deep paradigmatic differences often exist between EJ advocates and pro-establishment environmental actors (Table 8). The existence of multiple tensions begs the question if divisions are unsurmountable. Some preferences (e.g., market vs. non-market) could be difficult to reconcile; nevertheless, it would be premature to assume total incompatibility because mutual respect, education, and collaboration have seldom been afforded sufficient time or attention. To bridge divides, we recommend participatory research, discussed in the next section, as a starting point to find common ground and begin to bridge what has been identified as a “trust gap” [11].

Research helps define methods to improve distributive, procedural, and recognition justice, so our first recommendation is public, private, and philanthropic funding for collaborative research and advocacy involving EJ organizations, universities, NGOs, and legal advocates. Some university faculty successfully engage students in community-based learning projects while gathering research data alongside EJ organizations [26,75,76]. Second, it is important to increase engagement of scholars in advocacy and activism; if academics recognize and document the existence of injustices, the next step is to work for change. Third, since court cases have created important shifts in the energy sector [77], we recommend bridging grassroots, scholarly, and legal efforts. There is potential for additional litigation involving civil rights and social justice along with anti-pollution cases. Court settlements are one path to improve distributive justice, and can also create procedural openings by mandating involvement of impacted populations on future planning committees. Our final recommendation is to acknowledge and build upon intersectionality between distinct justice campaigns (e.g., labor, race, class, gender, etc.). We prioritize peer-to-peer exchanges and training programs to identify and build from connections between campaigns.

Taken together, the above recommendations could potentially help avoid what was identified in CJ conceptualizations as incongruence between scholarly theories, NGO policy recommendations, and grassroots movements’ perspectives [18]. The participatory research agenda outlined in Part 5 can assist in development of a shared vision.

## 5. A Participatory Energy Justice Research Agenda

Table 9 is a compilation of suggestions for future directions for EJ research, with participatory methods and geospatial tools highlighted as two important priorities. A promising goal is to use EJ screening to support proactive policies and decision-making to prevent damage rather than merely ameliorating harm after it has occurred [62].

Participatory research methods (1) build from local knowledge and access; (2) understand needs, priorities and concerns of community members; (3) refine and find new research questions; (4) enhance preparation, readiness, education, and leadership in partner organizations; (5) improve relevance and importance of research; (6) improve dissemination of results; and (7) establish a base to follow through with policy and action objectives [29,74,75,81,82].

Community-based participatory action research (CBPAR) often stimulates action following the documentation of problems [29,74,81]. For example, WE ACT members wore air monitoring gauges to document dangerous air pollution around Harlem bus terminals [29], and used their data to successfully lobby for the establishment of permanent air monitoring stations in locations of concern as well as to encourage the local government to switch to cleaner fuel for city buses. An additional benefit from this research was that the Harlem participants collecting air data were high school students, whose experience potentially encouraged them to pursue an advanced degree or a career in science. In a second case, in Richmond, California, a network of environmental and social justice organizations including CBE waged a successful campaign against Chevron’s expansion of oil refining in a neighborhood where 45% of adult residents living in the area for 15 years or more already suffer from asthma [26,74]. With the Northern California Household Exposure Study, a community-designed and implemented survey carried out in conjunction with academic partners, impacted populations produced data on health impacts they later would later share front of Richmond’s Planning Commission and a state court. In the end, Chevron’s proposal to expand was denied because their permit provided insufficient information on environmental and health impacts relative to the concerns community members raised [74]. Being involved in the exposure survey helped clarify the science of pollution for community members and provided important tools for impacted populations to defend themselves against one of the strongest economic actors in the state.

For positive action to follow from CBPAR, findings need to be distributed to all partners using accessible language and demonstrating respect for the contributions of each participant [81]. For marginalized groups, CBPAR exchanges can create direct practical results for recognition justice, while also strengthening local and collaborative capacity for procedural justice. Simultaneously, scholars who expand and improve community collaboration find opportunities to increase pedagogical and methodological breadth, publish procedures and findings, engage youth, and create long-term relationships with grassroots partners.

## 6. Conclusions

The above literature review and illustrative examples demonstrate important positive changes underway as well as on-going tensions. Concerns about the energy sector often emerge from disproportionate burdens, such as local clusters of ill health. Energy justice is often grounded in specific locations, but patterns of injustice tied to marginalization, discrimination, and racism transcend place.

While transitions away from fossil fuels may occur slowly, activists who pursue energy justice for their communities and beyond can become catalysts for social and ecological change. For example, LVEJO members give educational “toxic tours” in their neighborhood explaining the organization’s 20-year struggle to combat environmental racism and create a healthier community. Many EJ organizations publicize energy injustices in media outlets; in this way electricity users become aware of social and ecological impacts from their consumption, potentially broadening social networks for policy change or creating shifts in demand.

Place-based examples of equitable and beneficial energy interactions exist and are growing, but at the same time many decarbonization solutions involve risks for politically and economically marginalized populations. We highlight how transition away from fossil fuels creates broader social benefits when done with low-income and politically-marginalized groups in decision-making roles and positions of power [83]. Activists, advocates, and experts have laid out conceptual frameworks and guidance to begin to use energy justice as a decision-making tool [83,84,85]. We need empirical and long-term research to record noteworthy energy justice successes as well as to document and publicize on-going problems and conflicts. Where toxic pollution and maldistribution persists, participatory and collaborative research created with meaningful local engagement can help inform litigation, policy, advocacy, and activism to encourage energy justice transformations.

## Figures and Tables

**Table 1:** Current and proposed divestment strategies.

Strategy	Exclusion Target
1	All carbon reserves
2	All fossil fuels
3	Coal
4	Tar sands
5	The “Carbon Underground 200” ^1^
6	The “Filthy Fifteen” ^2^
7	Top EJ offenders
8	Non-compliant fossil fuel firms
9	Divest-invest

^1^ Top public companies in coal, oil, and gas based on potential GHG emissions from reported reserves. ^2^ The “Filthy Fifteen” are the highest carbon polluters, including Dominion Resources, Duke Energy, Arch Coal, and Peabody.

**Table 2:** Equity and justice potential from divestment.

Risks (R) and Opportunities (O)		Justice Type	Sources
R	--Reinvestment efforts are not as vibrant as divestment; there is the potential for job loss in fossil fuel dependent communities	Distributive	[4,22,46]
O	--Divestment movements encourage empowerment, particularly of students and youth activists	Procedural	[34,45]
R/O	--A small number of divestment campaigns incorporate struggles for race, class, sexual orientation, and gender equality	Recognition	[43,45,47,48]

**Table 3:** Equity and justice potential from carbon pricing.

Risks (R) and Opportunities (O)		Justice Type	Sources
R	--The increase in the prices of goods and services can cause an inequitable burden on low-income populations	Distributive	[15,51,52]
R	--Political interference creates low or inaccurate prices; exceptions create an unequal playing field	Procedural	[50,51]
R/O	--Carbon pricing improves intergenerational equity, but funds collected today may not be distributed for adaptation or to indemnify those suffering climate change-induced costs now	Distributive	[50,51,52,53]

**Table 4:** Equity and justice potential from cap-and-trade.

Risks (R) and Opportunities (O)		Justice Type	Sources
R/O	--Reductions in other harmful pollutants could occur at the same time that carbon is reduced, but co-pollutant reductions of certain toxins in particular locations are uncertain	Distributive	[15,53]
R	--Effective design is challenging; it is difficult to target positive social, health, and ecological outcomes; intensification of toxic hotspots is possible	Distributive	[55,56]
O	--There can be redistribution of funds to historically burdened areas or to expand research, development, and installation of low emission energy	Distributive	[53,57,58]
R	--Industry control over flexibility in a market-based system reduces the role of the state as well as opportunities for civic engagement	Procedural	[21]
R/O	--There has been some involvement of EJ and other social and environmental organizations in design and implementation; nonetheless, concerns about procedural justice continue	Procedural	[57,58,59,60,61]

**Table 5:** Equity and justice potential from renewable energy.

Risks (R) and Opportunities (O)		Justice Type	Sources
R/O	--There are extensive job training and employment opportunities, although these are not always accessible to low-income populations	Distributive	[21,22,65]
O	--Renewable energies can be engines of growth, innovation, and entrepreneurship; they can stimulate urban revitalization and “green recovery“	Distributive	[21,27]
O	--Declining costs make renewables more affordable	Distributive	[31]
R	--There is a rooftop solar access gap with uneven adoption	Distributive	[23,66]
O	--Grassroots organizations include renewable energy access and employment in broader social, economic, and environmental justice campaigns	All	[6,47]

**Table 6:** Equity and justice potential from energy efficiency.

Risks (R) and Opportunities (O)		Justice Type	Sources
O	--Changes are cost effective, so programs can have greater reach	Distributive	[67]
O	--There are opportunities for workforce development and employment; programs can contribute to urban revitalization	Distributive	[5,27,35]
R/O	--Efficiency reduces GHG emissions along with lowering utility bills, but renters could be left out	Distributive	[67,68]
R	--Upgrades are often inaccessible to low-income populations	Distributive	[69]
O	--Several grassroots organizations include energy efficiency in broader social and/or environmental justice campaigns	All	[see Table 7 below]

**Table 7:** Illustrative energy justice cases.

Name	Location	Synopsis
WE ACT	New York	WE ACT supports the Environmental Justice Leadership Forum on Climate Change and is working towards local energy democracy and climate resiliency with solar micro-grids
WDC Solar	District of Colombia	This was the first African American-owned solar manufacturing plant; It supports local job training and solar installations on city schools
510nano	North Carolina	A 1.4 megawatt solar farm developed on former cotton fields is the largest African American-owned; 70% of project builders were people of color
Energía	Massachusetts	This energy services company is owned by Nuestras Raices, Co-op Power, and Nueva Esperanza; Energía Worker Trust supports efficiency upgrades, healthy buildings and strong communities
Louisiana Green Corps	Louisiana	The Corp involves un- and underemployed youth, including those with intellectual disabilities, in weatherization and utility upgrades; Created in the wake of Hurricane Katrina, this community redevelopment initiative targets affordable housing
Green Impact Zone	Missouri	This community-run enterprise in a disadvantaged neighborhood; implemented a Smart Grid, renewable energy projects, energy efficiency upgrades, job creation and training programs, and more
Qualco Energy	Washington	Qualco is owned and managed by Native American Tulalip tribes in collaboration with a nonprofit and local farm owners; The project converts animal waste into biogas while reducing excessive nitrate loads in local waterways to restore salmon habitat
LVEJO	Illinois	LVEJO worked to shut down two coal plants in their neighborhood and seeks create renewable energy job training centers or green energy hubs

**Table 8:** Paradigmatic clashes.

Type	Example	Sources
Structure	--EJ movements’ decentralized, grassroots strategies contrast to hierarchical structures in many governmental, non-governmental, and private sector organizations; tensions frequently arise between administrative efficiency and broad participation	[21,42]
Scale	--The utilitarian ideal of the greatest good for the greatest number conflicts with special protections for burdened minority groups; tensions can emerge between global/national/state emission reductions and local rights and needs	[18,21,42]
Target	--Single-pollutant and single-industry controls do not create the systemic change sought in EJ and CJ movements; individualistic motivations can clash with collective or community solutions	[18,21,42,61,74]
Means	--State-centric or market-based programs limit power and agency of low-income actors; resources are prioritized for top-down versus bottom-up approaches	[18,21,51]

**Table 9:** A participatory energy justice research agenda.

Research Topics and Methods	Sources
--Promote community-based and participatory collaborations; advance multidisciplinarity and mixed methods	[29,58,74,76]
--Expand historicized and longitudinal geospatial analysis of demography, epidemiology, risk, etc.	[58,62,76,78]
--Improve data collection and refine modeling mechanisms; include cumulative exposures	[58,62,76,79]
--Identify policy responses to documented injustices; address root causes to improve community health, empowerment and resiliency	[18,79]
--Assess political economy of “just transition“ in specific places: identify EJ linkages between energy, climate, food, housing, zoning, education, jobs, resource access, contamination, etc.	[2,42,78,79,80]
--Increase cross-border, cross-cultural, and cross-movement activism; assure action follows from research	[42,80]
--Identify connections between consumption and production; use whole systems approaches for research and analysis	[9,10]

## Abbreviations

The following abbreviations are used in this manuscript:

AB 32	California Global Warming Solutions Act of 2006
CBE	Communities for a Better Environment
CBPAR	Community-based participatory action research
CJ	Climate justice
EJ	Environmental justice
EPA	Environmental Protection Agency
GHG	Greenhouse gas
LVEJO	Little Village Environmental Justice Organization
NGO	Nongovernmental organization
O	Opportunities
R	Risks
RPS	Renewable Portfolio Standard
UN	United Nations
U.S.	United States
WE ACT	West Harlem Environmental Action, Inc.
